# Opium Habit Cured by Galvanism

**Published:** 1879-09-20

**Authors:** William F. Hutchinson


### *OPIUM HABIT CURED BY GALVANISM.*

BY WILLIAM F. HUTCHINSON, M.D.

About the first of January of the present year, I was requested by Dr. O. C. Wiggin, of this city, to see with him a lady supposed to be suffering with cerebral congestion in an advanced stage.

A visit to Mrs. S. revealed the following history: Age 39; married; one child æt. six, and has had one miscarriage. Weight, about 150 pounds, and general appearance of contour and skin good. Patient kept up a low moaning, answering most of my questions intelligently, then relapsing into a semi-unconscious condition. Pulse 100, compressible. Temperature 99°; no loss of control of evacuations; conjunctivæ congested and pupils contracted closely; perspiration starts upon the smallest exertion, which also causes pain in abdomen and excites vomiting, which has lately become persistent, accompanied with intense thirst. Hands and feet cold, with shrivelled palms and plantar surfaces. No difference in temperature of head and axilla.

Ophthalmoscopic examination gave retinal and choroidal congestion, with venous enlargement, slight optic neuritis, and choked disk.

There was constant pain, and sense of fullness in frontal region.

The only family history that could be obtained bearing upon the case was the death of one sister a year ago, from acute brain inflammation, the remainder of the immediate family being still living and in good health; and the present condition appeared to be the culmination of six years of almost constant pain and general nerve exhaustion, following the birth of the child, aggravated by the subsequent miscarriage.

At this visit no suspicion was entertained by me of any opium habit, and the case was diagnosed as passive cerebral congestion, dependent upon general neurasthenia.

The next day Dr. Wiggin called and gave me the following additional items, which at once placed the case in its proper light, and gave the key to many of the symptoms before cited. After her confinement, which was a long and painful one, she suffered severely from wandering pains in back and hips, for which her attending physician at the time ordered tincture of opium applied externally, giving at the same time ten drops by the mouth, and the ground was broken for the building of the habit. The dose steadily increased until she came under the charge of Dr. Wiggin, some six months previous to my seeing her, when she was taking four ounces of laudanum daily internally, besides continuing external applications as before. Attempts were made to stop the pernicious habit, but it was too late for wise counsel to avail, and the usual cunning of opium-eaters procured for her the drug in spite of every effort of both husband and physician.

All forms of concurrent medication had been faithfully tried, but nothing was of use except the opium, to which it became absolutely necessary to resort occasionally, as, without it, the poor lady would arouse the neighborhood with agonizing screams and cries.

At this juncture, and as a forlorn hope, it was decided to essay galvanism, hoping that its great vitalizing power might aid in restoring tone to the exhausted nerve-centres. At my suggestion Collis Brown's chlorodyne was given in place of laudanum, and produced the same effect with an ounce per diem that the four ounces of the former had done.

Central galvanism was applied with a twenty-four cell Bartlett battery, using six cells from the cilio-spinal centre to the forehead with a downward current; then from the cervical vertebræ to the solar plexus with an ascending current, each lasting six minutes, or until the skin was thoroughly reddened under the negative carbon point. For the first few days applications were made morning and night. In a week the vomiting had ceased and consciousness returned, and the evening sitting was omitted. After a month the dose of anodyne was gradually decreased, but with every diminution the nausea returned, and nothing but a return to the old dose would avail. But her condition was very much improved. She slept better, the eyes were normal as to color, and the palms were no longer dry. At the close of the second month she was able to sit up, and the dose of anodyne was steadily cut down without the patient's knowledge, by adding to the chlorodyne a sufficient quantity of flavored treacle to replace each dose taken, until at that time an ounce would last three days.

Her general condition was greatly improved, and she began to take interest in her surroundings. In two months more she commenced to go out, and came to my office for treatment when I changed the current to the Siemens and Halske cabinet cell, which with its low tension and perfect capability of control, I regard as the ideal battery for central galvanism. There was no further trouble, and to-day, June 21st, the lady is quite well, attending to all her household duties, not having tasted opium in any form for seven weeks, and expressing unbounded delight at being free from the terrible habit which had so long been her master.

The *rationale* of the action of galvanism in this case is difficult to un derstand. When the circuit was closed over the superior cervical sympathetic ganglion, Dr. Wiggin and myself distinctly observed a sudden wavelike contraction of the distended retinal veins, which resumed their size in a few moments after the stimulus was removed. But, after some weeks' treatment, these veins became normal, and the intraocular congestion had disappeared *pari passu* with the cerebral symptom, and having repeatedly witnessed the same phenomenon in other cases, I am led to believe that the galvanic current has a direct tonic influence upon the vaso-motor system, which accounts for the occasional surprising results obtained in cases of cerebral congestion. With the advent of increased nerve circulation came an absolute horror for the drug, and it is not easy to know to what to attribute the increase of strength of will up to the point of totally dispensing with it of her own accord, unless it be to some change in mental power, due to increased nerve tone, the direct result of what I have before termed the vitalizing power of the galvanic current. Faradism was not at any time employed.

Dr. Wiggin gives full credit to the special treatment for the cure of the case.—*N. Y. Med. Record.*

---

**Cancer Treated by Eucalyptus.**—M. Luton, of Rheims, gave a patient with cancer, eucalyptus internally and locally, with good results—the cancer healing entirely.

